# Maximizing *Populus tremula* biomass conversion: synergistic pretreatment effects on sugars release and lignin recovery

**DOI:** 10.1186/s40643-026-01040-5

**Published:** 2026-04-15

**Authors:** Sharib Khan, Vahur Rooni, Daniel Rauber, Nikki Sjulander, Markus Gallei, Christopher W. M. Kay, Sabarathinam Shanmugam, Timo Kikas

**Affiliations:** 1 Chair of Biosystems Engineering, Institute of Forestry and Engineering, Estonian University of Life Sciences, Tartu, Estonia; 2 Polymer Chemistry, Saarland University, Campus C4.2, 66123 Saarbrücken, Germany; 3 Saarene, Saarland Center for Energy Materials and Sustainability, Saarland University, 66123 Saarbrücken, Germany; 4 Physical Chemistry and Chemistry Education, Saarland University, Campus B2.2, 66123 Saarbrücken, Germany; 5 London Centre for Nanotechnology, University College London, 17-19 Gordon Street, London, WC1H 0AH UK

**Keywords:** Biorefinery, Lignocellulosic biomass, Protic ionic liquid, Sugars, Lignin

## Abstract

The transition to a forest-based economy relies on sustainable alternatives that can efficiently convert renewable resources into chemical and material products. Sugar-centered wood fractionation processes, also called modern biorefineries, offer significant potential as a renewable precursor for the substitution of petrochemical derivatives. However, unlocking the full potential of advanced biorefineries requires optimized pretreatment strategies to valorize all biomass-side streams. This work evaluated the distinctive and connective applications of nitrogen explosive decompression (NED), protic ionic liquid (PIL), and alkaline pretreatments for the production of monosaccharides, oligosaccharides, and high value lignin from aspen (*Populus tremula*) biomass within a biorefinery context. Each pretreatment method uniquely influenced biomass fractionation, affecting the hemicellulose and cellulose dissolution, delignification, and the physicochemical properties of the remaining cellulose and lignin components. The findings indicate that PIL pretreatment alone yielded 56% monomeric sugars upon saccharification. Additionally, the combinatorial PIL-NED and Alkaline-NED pretreatment was superior for lignin removal, achieving 68% and 85% delignification while concurrently generating valuable side streams. Henceforth, integrated PIL-NED collaborative approach presents a considerable cost and efficiency advantage over conventional biorefinery pretreatments, offering a promising pathway for the co-production of monosaccharides and high-quality lignin.

**Graphical abstract:**

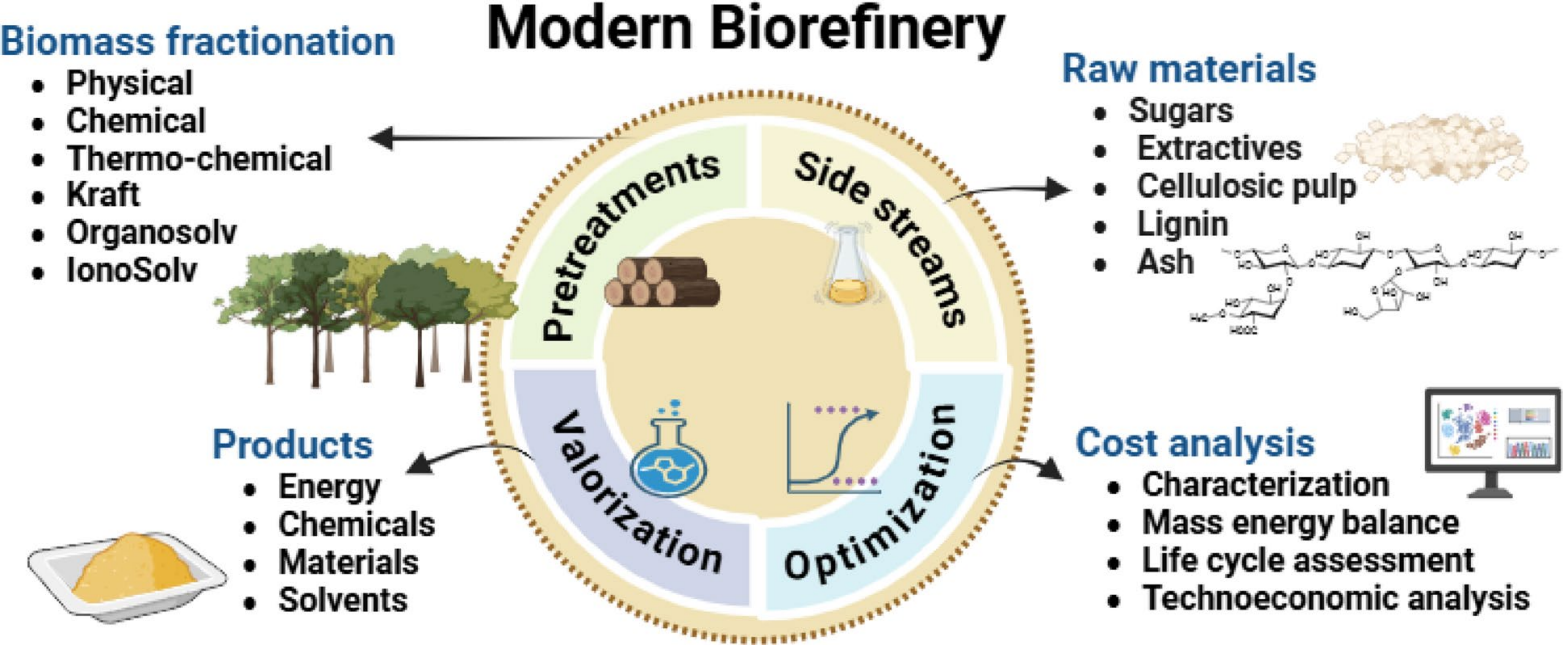

**Supplementary Information:**

The online version contains supplementary material available at 10.1186/s40643-026-01040-5.

## Introduction

The global economy remains fundamentally dependent on petroleum-derived products, including chemicals, plastics, and rubbers (Meys et al. 2021; Zibunas et al. 2024). A notable disparity exists in petroleum utilization, where transportation fuels consume over 70% of all petroleum, although their revenue is roughly equivalent to that generated by petroleum-derived materials and chemicals, which constitute only approximately 4% of the consumption (Dessbesell et al. 2018). This underscores the significantly higher value density of chemicals and materials compared with fuels. Consequently, the development of sustainable bio-based alternatives for chemicals and materials offers substantial potential for economic impact and climate mitigation (Tofani et al. 2023).

Biorefineries, which convert diverse second generation biomass feedstocks, such as wood and agricultural residues, into valuable bio-based raw materials, examples as cellulose, hemicellulose, and lignin, present a major opportunity (Liao et al. 2020). They align with sustainability objectives while offering resource-constrained nations a pathway to create economic value through the utilization of renewable resources. Hence, realizing the full potential of biomass necessitates a focus on producing high-value bio-based raw materials, such as lignin, that can eventually substitute crude oil-derived chemicals and materials. Thus, advancing the bioeconomy hinges on the development of cost-effective pretreatment technologies that utilize renewable feedstocks. However, significant commercialization bottlenecks still remain. Extensive research on sugar-based biorefineries has prioritized the optimization of oligosaccharide processing and sugar yields for biofuels, often relegating lignin to underutilized sidestreams (Abu-Omar et al. 2021). Generally, enzymatic hydrolysis following effective pretreatment is a well-established method for generating biofuels from lignocellulose (Vaaje-Kolstad et al. 2010; Angeltveit et al. 2024). However, the operating cost of enzymes remains a blockage to economic viability (Morizet-davis et al. 2025). Strategies for instance the production of plant-derived oils for food, feed, and oleochemicals using enzymatic hydrolysis and further fermentation of wood derived sugars, show promise for improving the economic feasibility of enzymatic processes (Losada et al. 2025). Ultimately, developing cost-effective pretreatments and integrating the valorization of sidestreams (hemicelluloses, lignin, extractives, and ash) are critical to meet the rapidly growing demand for bio-based materials (Rodrigues Gurgel da Silva et al. 2018).

Prominent emerging cost-effective biomass fractionation methods include Nitrogen Explosive Decompression (NED), aldehyde-assisted fractionation, alkaline pretreatment, and ionic liquid pretreatments (Baruah et al. 2018). These methods include mechanical (milling and grinding), chemical (alkali and ionic liquid), and physicochemical (steam and nitrogen explosion) processes that disrupt the recalcitrant lignocellulosic biomass. NED, for instance, is a cost-effective, commercially scalable, and environmentally favorable pretreatment (Rooni et al. 2021). It reduces the biomass particle size, dissolves hemicellulose, and disrupts cellulose crystallinity, offering advantages such as lower recycling costs, minimal energy requirements, and limited use of corrosive substances. Integrated pretreatment strategies designed to valorize all biomass sidestreams are essential for enhancing biorefinery economics and sustainability (Zang et al. 2020). This study addresses this imperative by evaluating the benefits, advantages, and limitations of integrated biomass pretreatment methods for the comprehensive valorization of sidestreams.

Nevertheless, lignocellulosic biorefinery research has extensively investigated herbaceous feedstocks, such as switchgrass and *Miscanthus*, for fractionation into sugars, furfural, ethanol, and lignin; studies utilizing woody biomass remain comparatively scarce (Bhatia et al. 2021; Chambon et al. 2021; Climent Barba et al. 2022). This gap is particularly evident in pretreatment strategies coupled with enzymatic hydrolysis in wood biorefineries. Furthermore, minimal research exists at the early technology readiness levels (TRL 1–2) specifically exploring integrated wood biorefinery concepts aimed at co-producing fermentable sugars, lignin, and cellulose pulp while systematically evaluating the distinct roles of various pretreatments (Tschulkow et al. 2020). This study directly addresses these critical gaps by investigating various wood fractionation processes used in biorefineries. The focus was on understanding the efficacy of enzymatic hydrolysis for sugars production, cellulose pulp yield, and lignin recovery. Following the pretreatments, this work also addressed mass energy balance and preliminary cost analyses at the laboratory scale that provided detailed economic viability and elucidated the functional contributions of different pretreatment methods within an integrated biorefinery framework.

## Materials and methods

### Materials

Raw Aspen (*Populus tremula*) hardwood biomass was collected from southern Estonia. The collected hardwood (includes bark), air-dried, chipped, grounded and milled to 1–2 mm, maintained at < 10% moisture, and stored in an air-tight container. The estimated compositional analysis of hardwood biomass (% of dry mass) is cellulose, 53.83 ± 0.10; hemicellulose, 16.90 ± 0.30; lignin, 13.89 ± 0.28; extractives, 7.80 ± 0.71; and others (ash, moisture and acids) 8.56 ± 0.24. All the chemicals were bought from Sigma-Aldrich (St. Louis, MO, USA) and heating experiments were conducted in a conventional heating oven.

### PIL synthesis

The protic ionic liquid (PIL) 2-hydroxyethyl(dimethyl)ammonium lactate ([N11H(2OH)][LAC]) was synthesized using commercially available reagents. 2-Dimethylamino-ethanol (> 99.5%) was sourced from Sigma-Aldrich (St. Louis, MO, USA), and racemic lactic acid (> 85%) was obtained from TCI Europe; A solution of racemic lactic acid (192 g, 2.24 mol, 1 equivalent) was prepared, to which 2-dimethylamino-ethanol (200 g, 2.24 mol, 1.05 equivalent) was added dropwise. The reaction mixture was incubated in a water bath for 2 h and then stirred for an additional 6 h. The product was isolated as a slightly yellow, viscous liquid in quantitative yield after removing excess amine and volatiles under high vacuum for 16 h as described previously (Khan et al. 2022).

### Biomass pretreatments

#### Nitrogen explosive decompression (NED) pretreatment

The Nitrogen explosive decompression (NED) pretreatment was applied to disrupt the biomass cell structure and expose cellulose fibrils for enzymatic hydrolysis. In a typical procedure, 100 g of dry biomass was mixed with deionized water in a pressure vessel (Parr Instruments) to form a watery paste, as described previously (Rooni et al. 2021). The reactor was closed with a customized pressure vessel cap and pressurized with N_2_ gas (10 bar). The pressure was regulated via manual ball valves and a manual pressure regulator, and controlled with two manometers, one on the cap of the pressure vessel and the second on the regulator of the N_2_ bottle. The reactor was then heated using a customized ceramic contact heater at 180 °C. The reactor temperature, monitored by a thermocouple, was regulated with a Unitronics controller. Upon reaching the target temperature, the system was cooled to approximately 80 °C before rapidly releasing the pressure through a valve. Finally, the pretreated samples were cooled below 50 °C in preparation for enzymatic hydrolysis.

#### Protic ionic liquid (PIL) pretreatment

For PIL pretreatment, 10 g of PIL and 3 g of untreated biomass (biomass-to-PIL ratio of 3:10 w/w) were used according to our previous work (Khan et al. 2022). Briefly, PIL pretreatment was performed in triplicate on biomass solids at 180 °C for 3 h. After pretreatment, the slurry was subjected to ethanol washes, followed by ethanol Soxhlet extraction. The concentrated PIL lignin mixture was transferred to 50 mL Falcon tubes, ~ 35 mL of hot deionized H_2_O was added, and the tubes were vortexed. Lignin was then precipitated from the PIL-lignin mixture with deionized H_2_O and recovery was done overnight using the drying vacuum oven at 45 °C for lignin mass-energy balances.

The recovered pulp was centrifuge at 4000 rpm for 10 min. The supernatant was collected, and the resulting pulp residue was repeatedly washed (at least four times) with 35 mL of hot deionized water, with centrifugation after each wash. The washed pulp was then freeze-dried for 48 h prior to the analysis. To determine the sugar content and composition, an aliquot of the combined wash supernatant was subjected to end-hydrolysis with 4% H_2_SO_4_ to break down oligosaccharides into monosaccharides.

#### Alkaline pretreatment

The process parameters of the alkali pretreatment, including NaOH concentration of 2 M, reaction temperature (90 °C), residence time (120 min), and biomass-to-liquid ratio (1:10 (w/v), were used to enhance lignin removal, as mentioned in our previous report (Cahyanti et al. 2023). Following pretreatment, the mixture was centrifuged (4000 rpm, 10 min). The supernatant was collected, and the biomass pellet was washed three times with 50 mL of distilled water, with the washings (liquid fractions) also collected. The combined supernatant and wash liquids were then acidified to pH 2 to precipitate the lignin. The precipitated lignin and the biomass solids were separated by centrifugation (4000 rpm, 5 min). Finally, both biomass and lignin fractions were individually washed to neutrality and dried at 60 °C for mass-energy balance.

#### PIL and alkaline assisted NED pretreatments

**Fig. 1:**
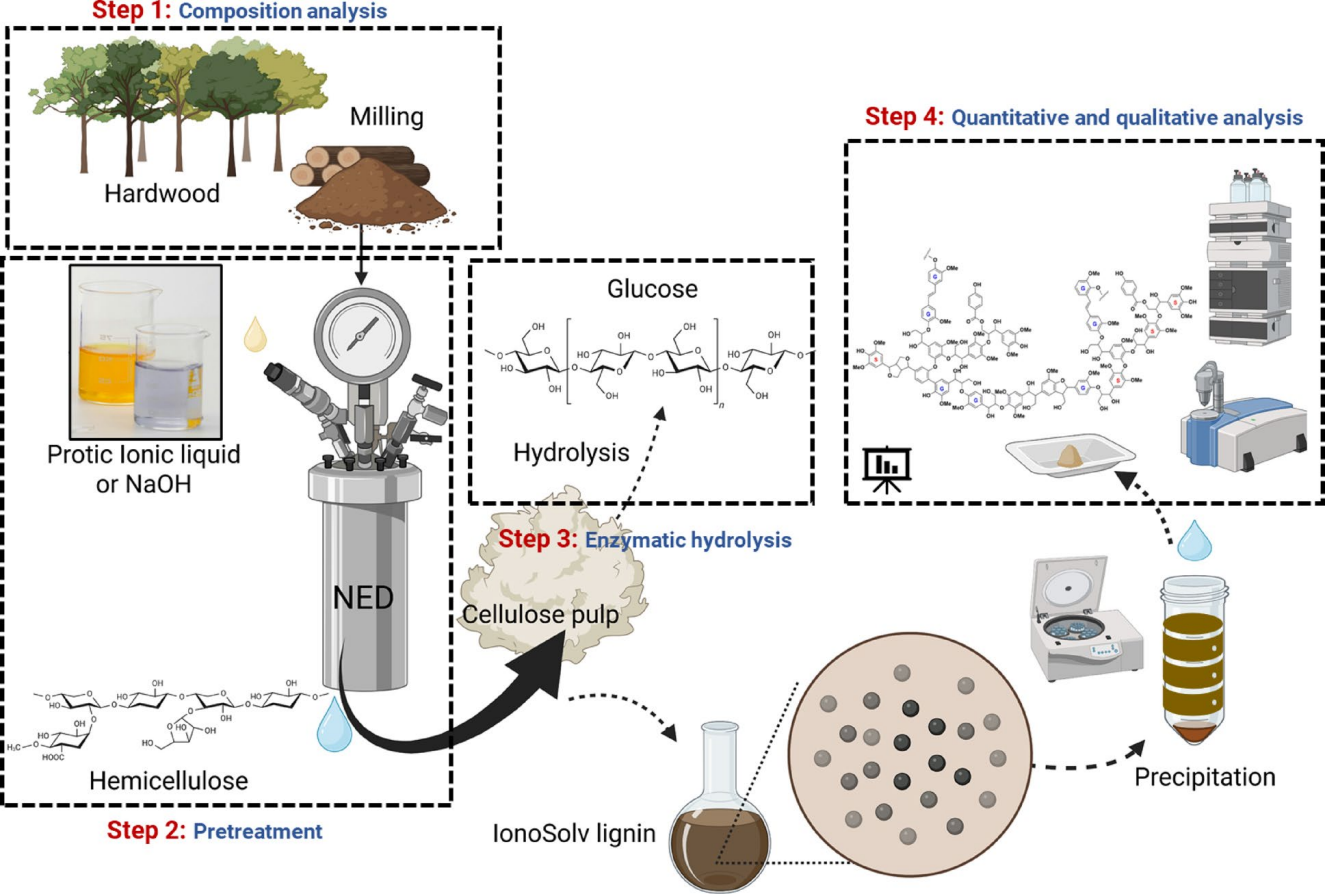
Schematic flowchart of the fractionation and conversion of aspen biomass (Steps 1–4) using PIL-NED or Alkaline-NED pretreatment. Co-products from the biorefinery process are extractives, furfural, hemicellulose, glucose, lignin, and cellulosic pulp

For PIL-assisted NED pretreatment (PIL-NED), 300 g of PIL and 100 g of untreated aspen biomass were used as shown in Fig. 1. Additionally, 100 ± 10 ml of deionized water was added until a watery biomass paste was obtained (biomass-to-PIL ratio of 1:4 w/w). The reactor was closed with a customized pressure vessel cap and pressurized with N_2_ gas at a pressure of 10 atm and a temperature of 180 °C. After reaching the target temperature (mentioned previously in NED pretreatment), the system was cooled to approximately 80 °C before rapidly releasing the pressure through a valve, the samples were cooled to a temperature below 50 °C, and the pulp-IL slurry was subjected to ethanol washes, followed by ethanol Soxhlet extraction, and further processed for fiber analysis.

For the alkaline-assisted NED pretreatment (alkaline-NED), 400 ml of [2 M] NaOH solution and 100 g of biomass were used as shown in Fig. 1 (step 2). The reactor was closed with a customized pressure vessel cap and pressurized with N_2_ gas at a pressure of 10 atm and a temperature of 180 °C. After pretreatment as mentioned previously in PIL-NED, the mixture was separated by centrifugation at 4000 rpm for 10 min. The supernatant was collected and further processed for fiber analysis.

### Enzymatic saccharification

Enzymatic saccharification was performed using the Suno™036 cocktail (Metgen Oy, Finland) on 1 g of untreated and delignified aspen biomass in a 100 mL screw-cap bottle. The related enzymatic activity towards different substrates was published elsewhere (Hämäläinen et al. 2021). The reaction was conducted in 15 mL of citrate buffer (0.005 M, pH 4.8) with an enzyme loading of 0.15 mL/g dry biomass. The mixture was incubated at 50 °C for 48 h in an orbital shaker at 220 rpm. After enzymatic hydrolysis, the hydrolysate was centrifuged at 4000 rpm for 5 min. Followed by the solid residue washing with 5 mL of fresh buffer, and the combined liquid fractions were collected for sugar analysis. The pH was verified using a Mettler-Toledo S220, Greifensee, Switzerland pH meter.

### Cost estimates of different pretreatment processes

In this study, several pretreatment methods were compared (NED, PIL, alkaline, PIL-NED, and alkaline-NED) for the bioprocessing of aspen wood and pretreated pulp into monomeric sugars and high-quality lignin at a small-laboratory scale. The methodology describing each step (process design, mass/energy balances and cost estimation) of the processes and considerations were based on recent available literature (Bhatia et al. 2021; Climent Barba et al. 2022; Yang et al. 2025). The different pre-treatment processes were divided into four sections (Fig. 1): biomass preparation, pretreatments, enzymatic hydrolysis with sugar recovery and lignin yield determination. Costs, such as the wood, PIL synthesis, pretreatments, electricity, water, anti-solvents, and enzymes, were gathered from the recent literature and are summarized in the later in the text. Capital investment and operational costs associated with sugars, lignin, PIL and EtOH recovery were excluded from the cost analysis.

### Analytical methods

#### Fiber analysis

The untreated and pretreated biomass moisture content was characterized with a Kern MLS-50-3D moisture analyzer. Its compositional analysis determining lignin, hemicellulose, and cellulose contents was performed using an ANKOM A2000 Fiber Analyzer (ANKOM, USA) according to standard detergent fiber protocols (utilizing Acid Detergent Fiber (ADF) and Neutral Detergent Fiber (NDF) solutions and 72% H_2_SO_4_). The ash content was determined based on the National Renewable Energy Laboratory NREL/TP-510-42622 procedure. Finally, mass and energy balances for the different pretreatment processes were developed using Microsoft Excel.

#### Scanning electron microscopy (SEM)

The different SEM images (at various magnifications) of the pretreated and untreated aspen wood were obtained using SEM Helios NanoLab 650 (FEI Company, Hillsboro, OR, USA) at an acceleration voltage of 10 KeV.

#### High performance size exclusion chromatography (HPSEC)

For different sugar analysis, the pretreated pulp was centrifuged twice at 10,000 rpm, and the supernatant was purified by filtration through a 0.2 μm PTFE filter (Thermo Scientific, USA). The purified samples were diluted (1:10 for glucose, 1:3 for acetic acid) and analyzed by HPLC (Shimadzu Prominence-i LC-2030 3D Plus, Shimadzu Corporation, Kyoto, Japan) with an RID-20 A detector. Glucose and xylose were separated using sequential Rezex™ RPM-Monosaccharide Pb + 2 and RHM-Monosaccharide H+ columns at 85 °C, with a detector at 60 °C as mentioned previously (Rooni et al. 2021). The mobile phase was degassed, deionized water at 0.8 mL/min, with a 20 µL injection volume and a calibration range of 50–2000 mg/L. Different acids were analyzed using a Rezex™ ROA-Organic Acid H+ column at 50 °C. The mobile phase was 5 mM degassed H_2_SO_4_ at 0.5 mL/min, with a 10 µL injection volume and a calibration range of 200 mg/L to 20 g/L.

The molecular weight distribution of the extracted lignins was determined using the same Shimadzu Prominence-i LC-2030 3D Plus system. The system was equipped with a PSS MCX column set (1000 and 100,000 Å), a pre-column, and a UV detector (λ = 280 nm). Lignin samples were dissolved in 0.1 M NaOH (5 mg/mL) and eluted isocratically with the same solution at 0.5 mL/min, with a 20 µL injection volume. Relative molecular weights were calculated against polystyrene sulfonate sodium salt standards (1.1–100 kDa) as describe previously (Khan 2025).

#### FTIR

FTIR spectroscopy with attenuated total reflection (ATR) was employed to characterize the surface functional groups of different extracted lignins. The analysis was performed on a Perkin Elmer Spectrum (BXII spectrometer, Waltham, MA, USA) recording spectra from 4000 to 600 cm^−1^ with a resolution of 4 cm^−1^ and averaging 16 scans per sample was recorded.

## Results and discussion

### Synergistic effects of different pretreatment processes on biomass fractionation and component recovery

**Fig. 2:**
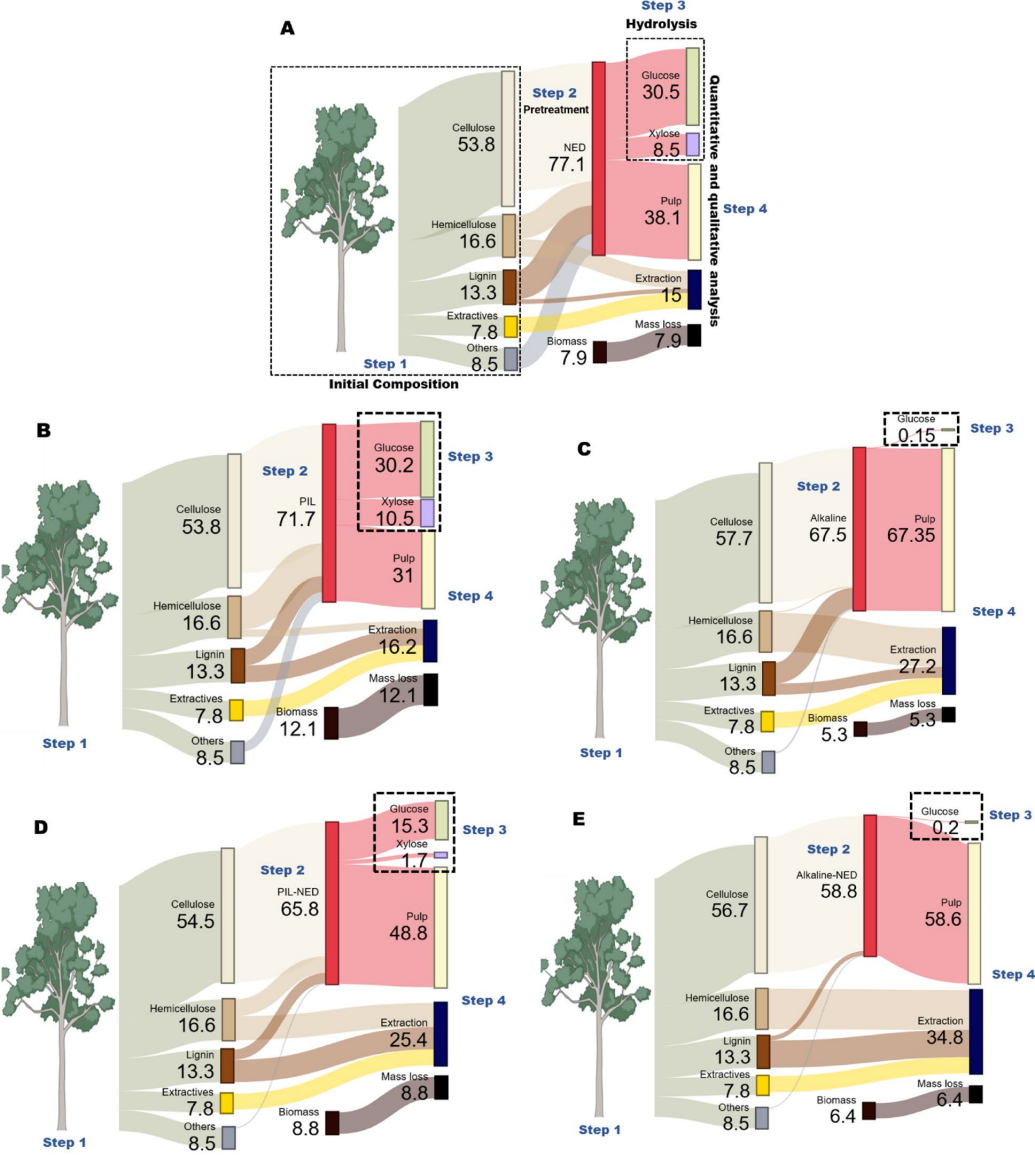
Mass balance flowcharts for (**A**) NED, (**B**) PIL, (**C**) Alkaline, (**D**) PIL-NED, and (**E**) alkaline-NED pretreatments in four major steps. Step 1: Milling, grinding and initial composition analysis of aspen biomass; Step 2: Pretreatment of the biomass and its extraction of different components; Step 3: Enzymatic hydrolysis of pretreated pulps; Step 4: Quantitative and qualitative characterization of extracted, hydrolyzed and loss biomass components

**A**–**E** compares the component recovery and mass losses across different pretreatment methods. NED, a physio-thermal pretreatment, disrupts structural components via high-temperature, pressurized N_2_ gas, initiating autohydrolysis and enhancing cell wall permeability to dissolved nitrogen (Rooni et al. 2021). This process resulted in a partial biomass loss (7.9%) during fractionation (**A**). 

PIL pretreatment is a thermo-chemical pretreatment that cleaved numerous bonds in the biomass fractionation process whilst extracting hemicellulose, and lignin. Efficient hemicellulose recovery is vital for this process economics, while the cellulose and some lignin fractions remained in the solid pulp (Brandt et al. 2013). The results in Fig. 2B show that PIL pretreatment can extract hemicellulose, lignin, and extractives while undergoing heavy biomass losses (12.1%) during processing, higher than the NED pre-treatment.

Alkali pretreatment can be used to efficiently treat graminaceous feedstocks because of its less severe pretreatment conditions compared with traditional pulping. Obtaining lignin from second generation biomass sources after NaOH pretreatment may require harsher conditions that risk altering the lignin structure (Xu et al. 2020). The alkaline conditions used in this study were not harsh; However, severe conditions were followed in alkaline-NED pretreatment to understand the role of hemicellulose and lignin extraction as showed in Fig. 2C and E. Similarly, increasing the temperature under alkaline conditions leads to lignin condensation with hemicellulose, as suggested in previous literature (Koistinen et al. 2025). However, under reasonable conditions, the alkaline process was able to extract almost 95% of hemicellulose, while delignification percentage was 34% with 5.3% loss in biomass (Fig. 4 and Supplementary Information Table S3). Unfortunately, hemicellulose recovery data for the different processes are not available in Table S2 and were excluded from the mass balance.

The most interesting results in terms of component recovery and recovered pulp percentages were achieved during PIL-NED and alkaline-NED pretreatments (Fig. 2D and E) mass losses 8.8 % and 6.4 %, respectively. Based on the results both the PIL and alkaline assisted NED pretreatments yielded higher recovered pulp as well as lignin yields. Previously, Gominho et al. (2019) and Tschulkow et al. (2020) have described the effect of different pretreatment methods to improve biomass fractionation and give an overall integrated techno-economic assessment of an integrated biorefinery process. Also, Schutyser et al. (2018) describe in detail the interplay of lignocellulose in the field of biomass fractionation and its industrial implementation. As observed in this study, the combination of PIL, alkaline assistance, and physical pretreatment is crucial for effective biomass fractionation, resulting in higher process efficiency and yield. The major advantage of PIL integration as PIL-NED pretreatment is the solvent recovery process, which accounts for the solvent being released after vacuum distillation, which can then be reused in an industrial system (Verdía Barbará et al. 2025).

The final masses of the solid pulp remaining after enzymatic hydrolysis varied between the samples. The alkaline pretreatment resulted in the highest pulp yield (~ 67.3 g), followed by alkaline-NED (~ 58.6 g), PIL-NED (~ 48.8 g), NED (~ 38.1 g), and PIL (~ 31 g) treatments. Lower yields of pulp indicate a more effective removal of sugars and other components (xylan, extractives, and lignin). While PIL and NED pretreatments individually converted a similar amount of glucan to monomeric glucose and xylose (~ 40.5 g), the combined PIL-NED method was particularly less effective, producing 17 g of monomeric sugars, whereas the alkaline and alkaline-NED method showed no significant sugar removal. A critical economic advantage of NED and PIL-based pretreatments is their integrated solvent recovery system, hemicellulose extraction, and potential lignin valorization without introducing chemical impurities (Nair et al. 2023). These results demonstrate that selecting an effective pretreatment is paramount, as it directly dictates the downstream processing efficiency, final sugars yield, and overall economic sustainability of biorefinery operations (Schutyser et al. 2018).

Recently Zhou et al. (2025) describe an environmentally friendly ternary deep eutectic solvent (DES) system for the efficient delignification of hardwood biomass and the selective isolation of high-performance cellulose. They achieved synergistic interactions among DES components which enabled effective lignin removal under mild conditions (130 °C, 90 min) whilst significantly reducing cellulose degradation. Similarly, Sun et al. (2024) proposed a biphasic system containing water-soluble DES and cyclopentyl methyl ether to treat hardwood for furfural production, extracting lignin and enhancing cellulose enzymatic hydrolysis. Future research would allow to employed such system for the PIL-NED pretreatment.

**Fig. 3:**
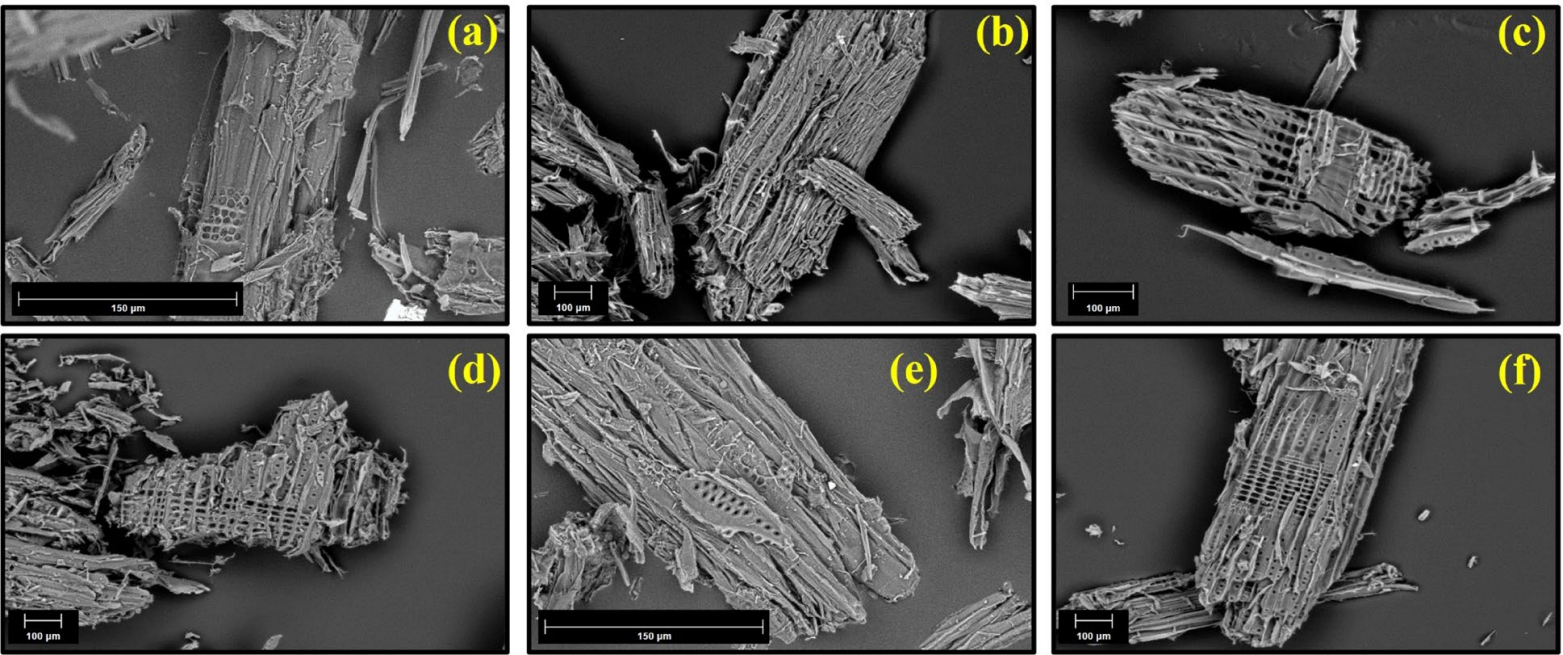
SEM analysis of aspen biomass before and after pretreatment: **a** Raw aspen biomass; **b** NED-pretreated aspen biomass; **c** PIL-pretreated aspen biomass; **d** PIL-NED pretreated aspen biomass; **e** alkaline-pretreated aspen biomass; **f** alkaline-NED pretreated aspen biomass

Moreover, to observe the physical changes SEM images before and after the pretreatments were used to visualize the effects of the different pretreatment methods on the biomass structure. Figure 3 illustrates the effects of pretreatment on the biomass particles. Figure 3a illustrates the untreated milled biomass, characterized by distinct individual fibers and surface fissures on larger particles, likely resulting from mechanical abrasion during size reduction by the chainsaw and grinder. Images in Fig. 3b–f were obtained following NED, PIL, PIL-NED, alkaline, and alkaline-NED pretreatments. These images indicate that the various pretreatment methods exerted a comparable impact on biomass integrity, while pretreatment at 180 °C (Fig. 3b–f excluding e resulted in more pronounced fiber disintegration. Similarly, at 90 °C (Fig. 3e), initial fissures between individual fibers became evident. This observation indicates that, in addition to temperature, both pressure and the catalyst significantly influence the efficacy of the pretreatment (Schutyser et al. 2018). Moreover, at higher temperatures and pressures, lignin functions as a binding material and softens, thereby facilitating the separation of fibers (Vanholme et al. 2010). Consequently, the porosity and specific area of pretreated biomass increased, leading to an increase in the efficiency of biomass fractionation, as shown in Supplementary Table S1.

The SEM results, while not offering a detailed analysis of the biomass fractionation or pretreated surface morphology, indicated that the pretreatment methods disrupted the aspen cell wall structure. For instance, the pretreated biomass has a lower ash content however considerable number of extractives, sugars, and inhibitors, which are mainly composed of polar compounds extracted using ethanol and water (Supplementary Table S1 and S2). In comparison, the untreated biomass displayed a highly ordered, intact structure characterized by a smooth surface and a compact, three-dimensional fiber network. Moreover, increasing the pretreatment temperature and pressure after achieving the threshold parameters would not severely increase the product yield, as previously discussed by Weerachanchai et al. (2012).

### The impact of inhibitors on the saccharification of pretreated biomass

To further investigate the enzymatic hydrolysis efficiency, the cellulose-enriched pretreated pulp was hydrolyzed using a commercial cocktail of enzymes (Suno^TM^036) that selectively digests the amorphous regions of cellulose to release monomeric sugars (glucose, xylose, arabinose, mannose, and cellobiose) while keeping the more recalcitrant crystalline cellulose regions. Supplementary Table S2 shows the glucan digestibility profiles to glucose, xylose, arabinose, mannose, and cellobiose after 48 h of enzymatic hydrolysis. The maximum glucose release from the untreated aspen was ~ 5% after 48 h, reflecting the low glucan digestibility of the aspen biomass. While ~ 77% and 71% of the obtained pretreated pulp after NED and PIL pretreatment was hydrolyzed into glucose and xylose after 48 h, ≤ 30% of glucose was released for NED and PIL within the same time interval. Surprisingly, the glucose yields for PIL-NED were lower than those for NED-pretreated solids by a factor of ~ 2 after 48 h (Fig. 2).

Unfortunately, the alkaline pretreated pulp and alkaline-NED pretreated pulp did not produce sugars, likely due to factors such as pH, inhibitors, presence of lignin, or smoother and more accessible surface area of the pretreated pulp (Momayez et al. 2022). (Gong et al. 2021) acknowledges the role of lignin in enzymatic hydrolysis inhibition whilst blocking lignin using peanut protein (PP), promoting the effect of PP on enzymatic hydrolysis. Similarly, the significantly higher enzymatic hydrolysis observed for the PIL and PIL-NED pretreated biomass compared to the alkaline and alkaline-NED pretreated pulp, despite greater lignin removal in the latter, highlights that saccharification efficiency is governed by a complex interplay of factors beyond simple delignification metrics. The primary reasons are the severity of the conditions and pseudo-lignin formation, as discussed in several studies (Qin et al. 2016; Gong et al. 2021). Moreover, harsh alkaline or pretreatment conditions can degrade solubilized carbohydrates into recalcitrant pseudo-lignin, which re-deposits as a potent enzyme barrier, whereas PIL conditions typically avoid these degradation pathways (Wang et al. 2025); Gschwend et al. 2019). Hence, the critical distinction lies in the properties of residual lignin. Previous studies have also suggested that alkaline pretreatment often leaves a lignin rich phenolic hydroxyl groups in pretreated pulp, which strongly and irreversibly binds cellulases (Qin et al. 2016). In contrast PIL treatment chemically modifies or passivates lignin, drastically reducing its non-productive affinity for enzymes (Brandt et al. 2013). This inherent inhibition in alkaline pulp also raises the issue of enzyme dosage sufficiency, as a standard loading may be rapidly sequestered by inhibitory surfaces, making effective hydrolysis economically unfeasible without prohibitively high enzyme use (Gong et al. 2021; Shi et al. 2025; Pari et al. 2025). In contrast, the more accessible and less inhibitory PIL-pretreated substrate allows for efficient enzyme action at standard loadings. Finally, the common practice of adding sulphite or acid during alkaline pretreatment, while improving delignification, can sometimes exacerbate issues by introducing sulfonated lignin or acid-induced precipitates that further complicate the surface chemistry (Zhong et al. 2022; Wang et al. 2024; Shi et al. 2025). Consequently, this study acknowledges higher molecular weight lignin’s extracted from PIL-NED and alkaline-NED processes, where higher severity conditions left high molecular weight lignins compared to solely PIL and alkaline based extracted lignins. Thus, we acknowledge that PIL creates a superior substrate by minimizing inhibitory by-products, passivating residual lignin, and maximizing cellulose accessibility, leading to disproportionately higher hydrolysis yields even with lower absolute lignin removal.

Interestingly, Guigou et al. (2019) acknowledges two consecutive treatment steps to hardwood sawdust in enhancing enzyme accessibility to cellulose. The first treatment step assayed was autohydrolysis (170 °C, 40 min) followed by the soda pulping (155 °C, 90 min). The results also showed that the efficiency of the enzymatic hydrolysis was higher than 70% in the case of an additional soda pulping while only autohydrolysis led to efficiencies lower than 60%. Altogether, Cabrera et al. (2016, 2025) and Xu et al. (2017) describe the role of hemicellulose recovery of the in wood, by a green liquor extraction stage prior to kraft pulping of hardwoods and recycled ionic liquids as green solvents for sustainable biomass pretreatment. A promising direction for future work involves the selective removal of hemicelluloses to improve enzymatic digestibility. It is important to note that while higher enzyme loadings can further increase sugar yields, they concurrently raise processing costs, significantly impacting the overall techno-economic feasibility.

Altogether, the reduced hydrolysis rate observed under alkaline conditions may also be attributed to the rigid structural properties of the cellulose-enriched pulps, as suggested by Penín et al. (2019). Enzyme deactivation or substrate saturation effects are unlikely to be contributors, given the relatively low Suno^TM^036 dosage (15% v/w enzyme-to-pulp ratio). The results indicate that sugar yield is directly linked to the conversion of crystalline cellulose to its amorphous form. The negligible glucose and cellobiose released from alkaline and alkaline-NED pretreated solids imply that these methods preserve the native crystalline structure and fiber orientation, rendering the cellulose surface inaccessible (Koistinen et al. 2025). These results also confirm that NED and PIL-NED pretreatments have similar synergy on the properties of the recovered pulp, making the pretreated pulps potentially suitable for saccharification and various biochemical applications (Angeltveit et al. 2024; Losada et al. 2025). Moreover, these hydrolysis results do not provide an industrially relevant target, the primary goal of these initial experiments was to determine the amount of glycan that could be converted to sugars for downstream fermentation. Additionally, trace amounts (≤ 5%) of PIL likely co-precipitated with the recovered cellulose owing to the intercalation between cellulose fibrils, despite excessive washing of the pulp (Table 1). Previous studies have reported that a residual PILs concentration ≥ 5% can inhibit cellulase activity and hinder downstream fermentation processes (Bhatia et al. 2021). Given the low enzyme dosage (Suno^TM^036) and low pulp loading employed in this study, it is unlikely that pulp digestibility was inhibited by enzyme-related factors or residual PIL, as shown in Table 1. Future research should also focus on the effects of reduced PILs concentrations and enzyme loading, with an emphasis on optimizing compatibility across bioethanol production stages, particularly in enzymatic hydrolysis.

**Table 1:** Inhibitory compounds formed during biomass pretreatment

Conditions	Furfural (%)	HMF (%)	Lactic acid (%)	Acetic acid (%)	Formic acid (%)
NED pretreatment	1.74 ± 0.13	0.25 ± 0.02	0.31 ± 0.04	2.61 ± 0.24	0.37 ± 0.07
PIL pretreatment	0.03	nd*	3.49 ± 0.14	nd	nd
NED-PIL pretreatment	nd	nd	4.86 ± 0.03	nd	nd
Alkaline pretreatment	0.02	nd	nd	nd	nd
Alkaline-NED pretreatment	nd	nd	nd	nd	nd

*nd, not detected

### Quantitative and qualitative lignin-related properties

**Fig. 4:**
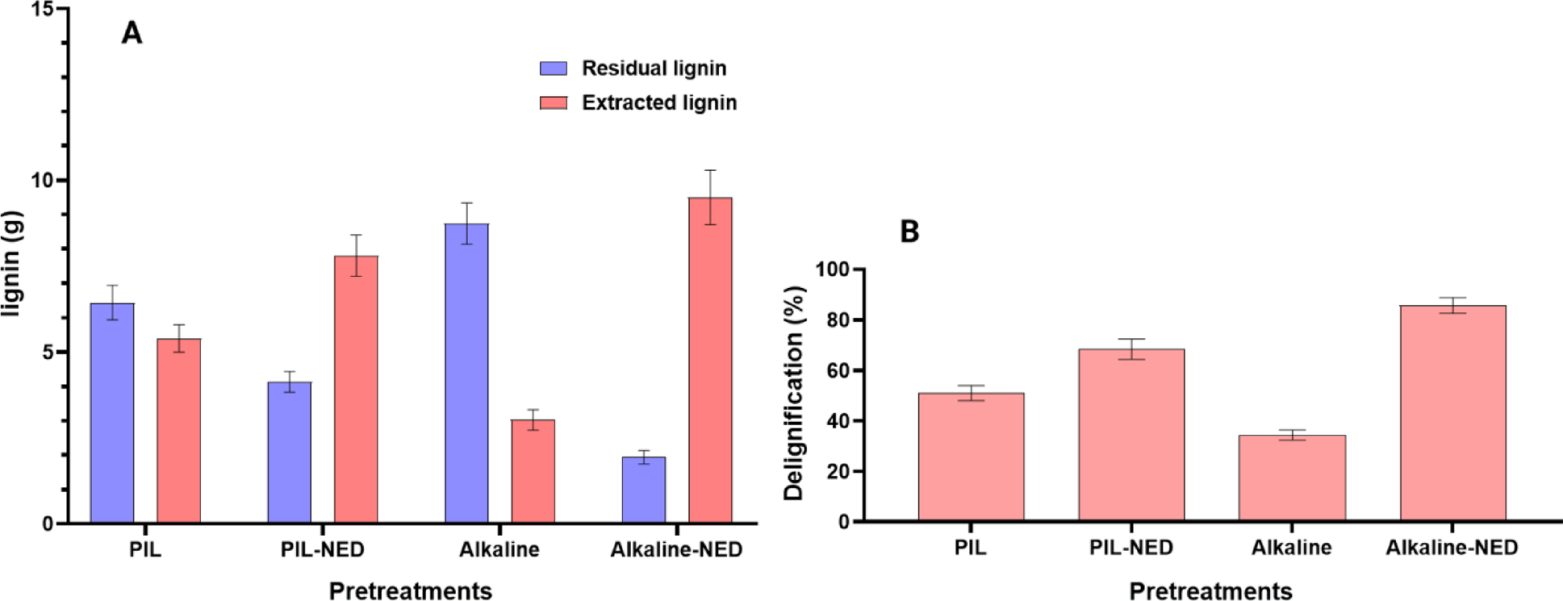
**A** Comparison of residual lignin content in different pretreated solids with yield of lignin precipitated from the liquid streams. **B** Extent of delignification achieved by the different pretreatment methods

We determined the delignification percentages of different pretreatment processes by analyzing the composition of the recovered pretreated biomass. The extracted lignin yield was calculated from the mass of lignin precipitated after separation (Supplementary Table S3). The delignification of aspen biomass by PIL, PIL-NED, alkaline, and alkaline-NED pretreatments was determined by estimating the residual lignin left in the biomass before and after pretreatment on a dry weight basis, and calculated based on a previously reported equations (Khan 2025). Similarly, lignin recovery from the different pretreatments was determined based on the amount of lignin yielded from the precipitation relative to the initial lignin content in the raw biomass (Fig. 4A).

The delignification efficiency was significantly higher in PIL-NED and alkaline-NED pretreatments, reaching approximately 68% and 85%, respectively, compared to 51% and 34% achieved through PIL and alkaline pretreatments (Fig. 4B). This enhanced lignin removal is attributed to the catalytic role of PIL (Chambon et al. 2021) and alkaline agents (Singh et al. 2019), which facilitate lignin extraction during biomass fractionation. Previous studies have highlighted the efficacy of catalysts and organic solvents in selectively solubilizing lignin, supporting the concept of these assisted methods as “lignin-first” approaches to biomass processing (Ragauskas et al. 2014); Balakshin et al. 2021).

**Fig. 5:**
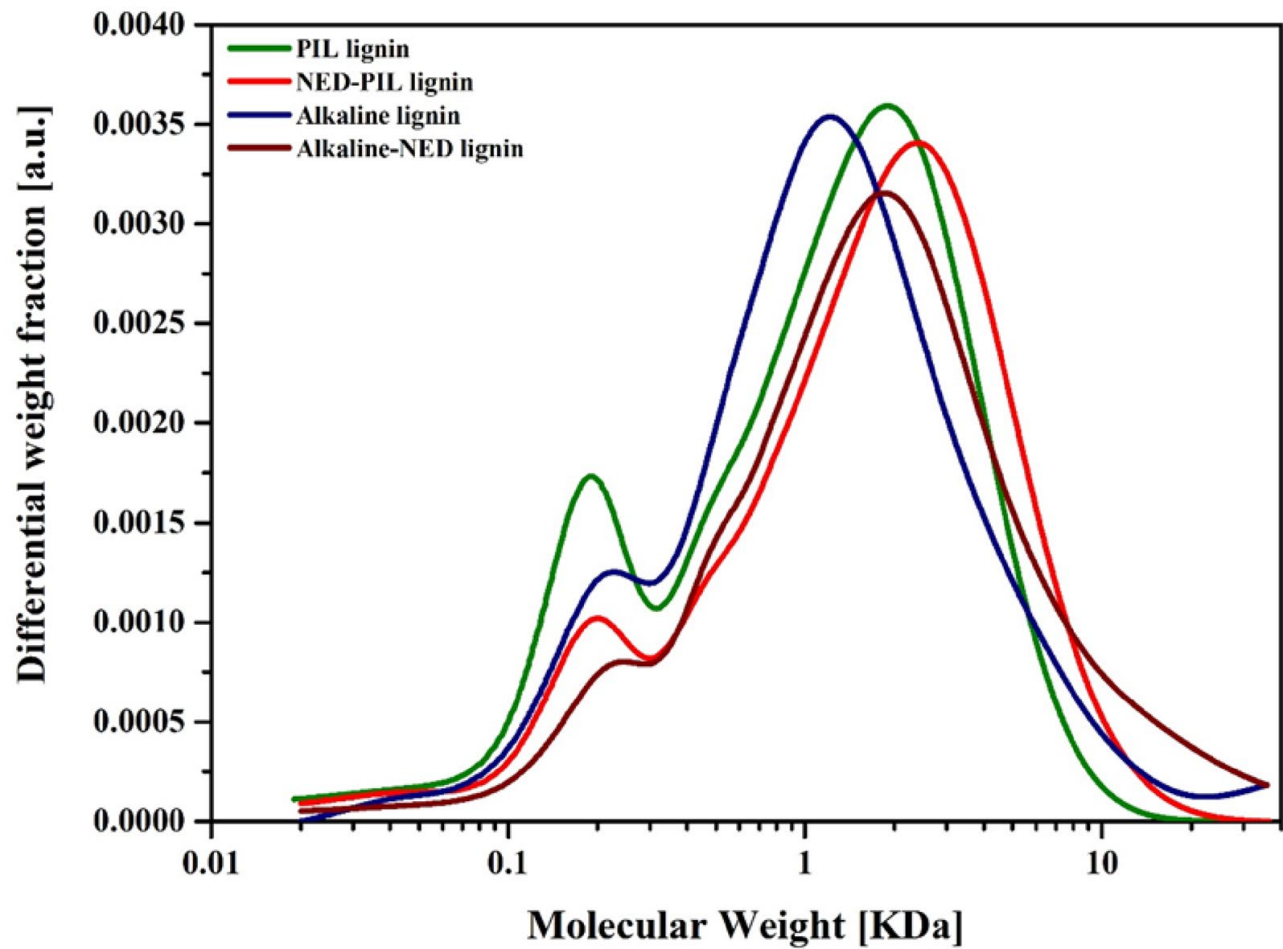
Size exclusion chromatogram of different extracted lignins dissolved in 0.1 M NaOH. Eluent flow rate at 0.5 mL min^−1^ using polystyrene sulfonate sodium salt standards ranging from 1100 to 100,000 Da

Furthermore, the changes in the weight-averaged molecular weight (M_w_) and number-averaged molecular weight (M_n_) of the extracted lignin were measured using High performance size exclusion chromatography (HPSEC) LabSolutions GPC software. The relative molecular weight distribution method was used to acknowledged the changes in the sizes of different lignin networks. The M_w_ values of PIL-NED and alkaline-NED were 2978 and 4426 g mol^−1 ^, respectively. Notably, the molecular weight distribution curves of PIL and alkaline extracted lignins were less than those of the assisted pretreatments, that is, 2086 and 3005 g mol^−1^ (Table 2). Similarly, the chromatograms of different lignins (Fig. 5) show the same characteristics, with assisted pretreatments having higher relative molecular weights than individual pretreatments. These differences in molecular weight resonate to the lower carbohydrate content in PIL and alkaline-extracted lignin. Previously, many authors, including us, have reported an increase in lignin molecular weight due to the caramelization of sugars with lignin at higher pretreatment temperatures (Khan et al. 2025; Liu et al. 2021, 2022; Rodrigues et al. 2021). Furthermore, Giummarella et al. (2019), Lawoko et al. (2005) and Tarasov et al. (2018) described the role of lignin-carbohydrate complexes (LCC), which are lignin moieties in deciduous species that are bound to hemicellulose or cellulose. Therefore, these LCC linkages within lignin substructures reduce hydrolysis efficiency and biomass digestibility while increasing the molecular weight (M_w_) of lignin during extraction. Consistent with this mechanism, the PIL-NED pretreatment decreased enzymatic hydrolysis, whereas Alkaline-NED showed negligible hydrolytic activity.

**Table 2:** Average molecular weights of different extracted lignins

Extracted lignins	M_*n*_ (g mol^−1^)	M_w_ (g mol^−1^)	PDI
PIL extracted lignin	555	2086	3.76
PIL-NED extracted lignin	748	2978	3.98
Alkaline extracted lignin	685	3005	4.39
Alkaline-NED extracted lignin	928	4426	4.77

**Fig. 6:**
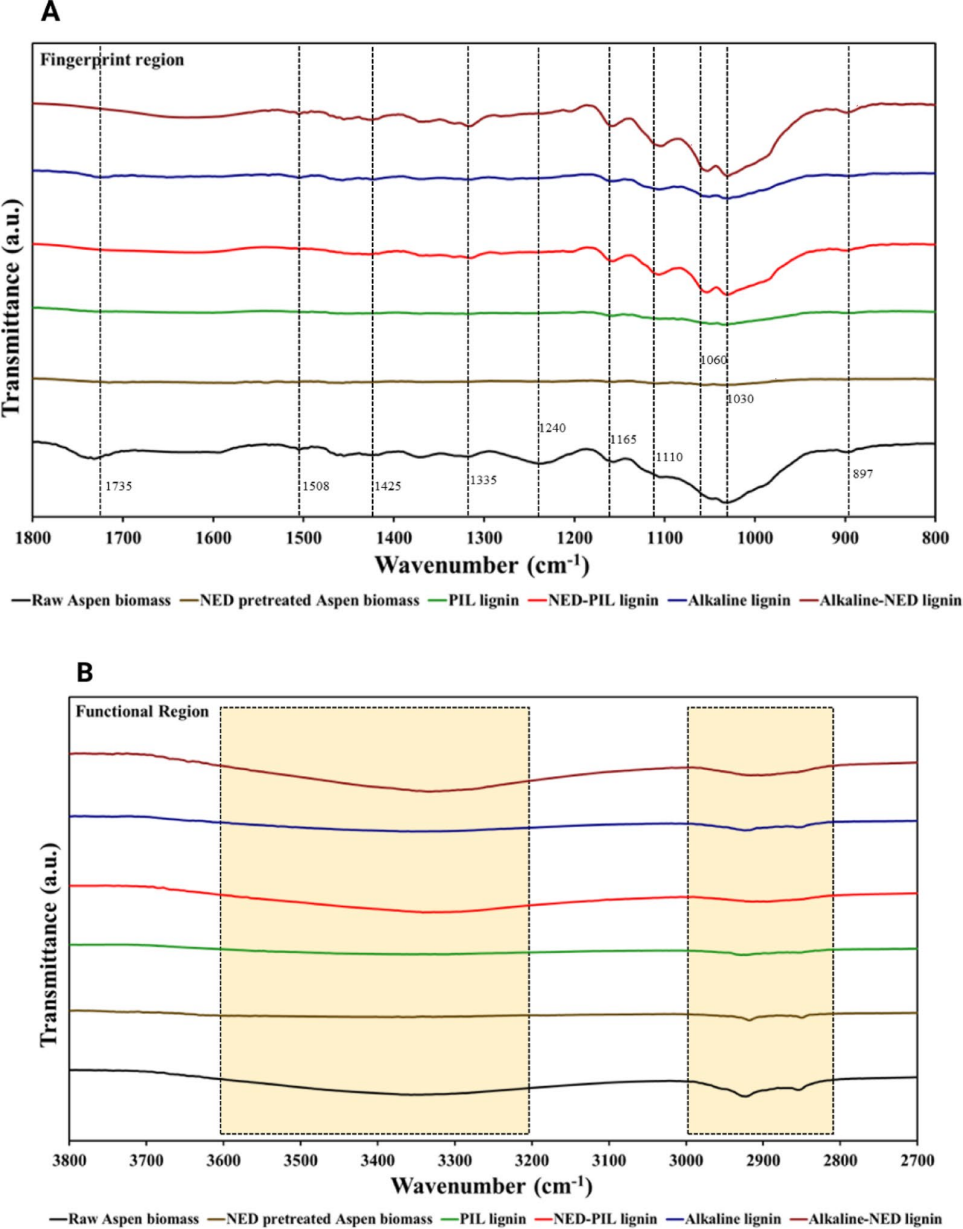
ATR-FTIR spectra of different extracted lignins

ATR-FTIR spectra of lignins extracted via different pretreatment methods exhibited similar overall trend as mentioned in HPSEC, with notably higher signal intensities observed in the PIL-NED and alkaline-NED (Fig. 6). Broadly, the ATR-FTIR spectra were divided into two characteristic regions: the “fingerprint” region (800 to 1800 cm^−1^) and the functional region group from (2700 to 3800 cm^−1^). In the fingerprint region (Fig. 6A), C–O stretching vibrations and specific absorption bands can be attributed to the lignocellulosic components: peaks at 1595, 1508, and 1270 cm^−1^ are characteristic of lignin (Kurian et al. 2015); bands at 1460, 1425, 1335, 1220, and 1110 cm^−1^ are associated with carbohydrates together with the lignin; and peaks at 1735, 1375, 1240, 1165, 1060, 1030, and 897 cm^−1^ correspond to carbohydrates (Wijaya et al. 2025); Zhang et al. 2017).

Similarly, reduced C–H stretching vibrations (2994–2806 cm^−1^) was consistent with earlier reports and key spectral changes include the disappearance of the 1740 cm^−1^ and 1238 cm^−1^ bands, both of which were attributed to hemicellulose following all pretreatments (Zhang et al. 2017). The samples subjected to NED and PIL pretreatment showed a marked reduction in the hemicellulose peak at 1238 cm^−1^. Meanwhile, in the PIL-NED and alkaline-NED samples, a reduction in the 1595 cm^−1^ lignin-associated band was observed, indicating more extensive lignin disruption relating to C=C skeletal vibrations of the benzene ring (Liu et al. 2024). Furthermore, the pretreatments also induced a slight decrease in the amorphous (897 cm^−1^) and the crystalline (1423 cm^−1^) regions of cellulose (Punia Bangar et al. 2023). Henceforth, elevated signal intensities were observed in the functional group regions of the PIL-NED and alkaline-NED lignin samples (Fig. 6B).

### Cost analysis

**Fig. 7:**
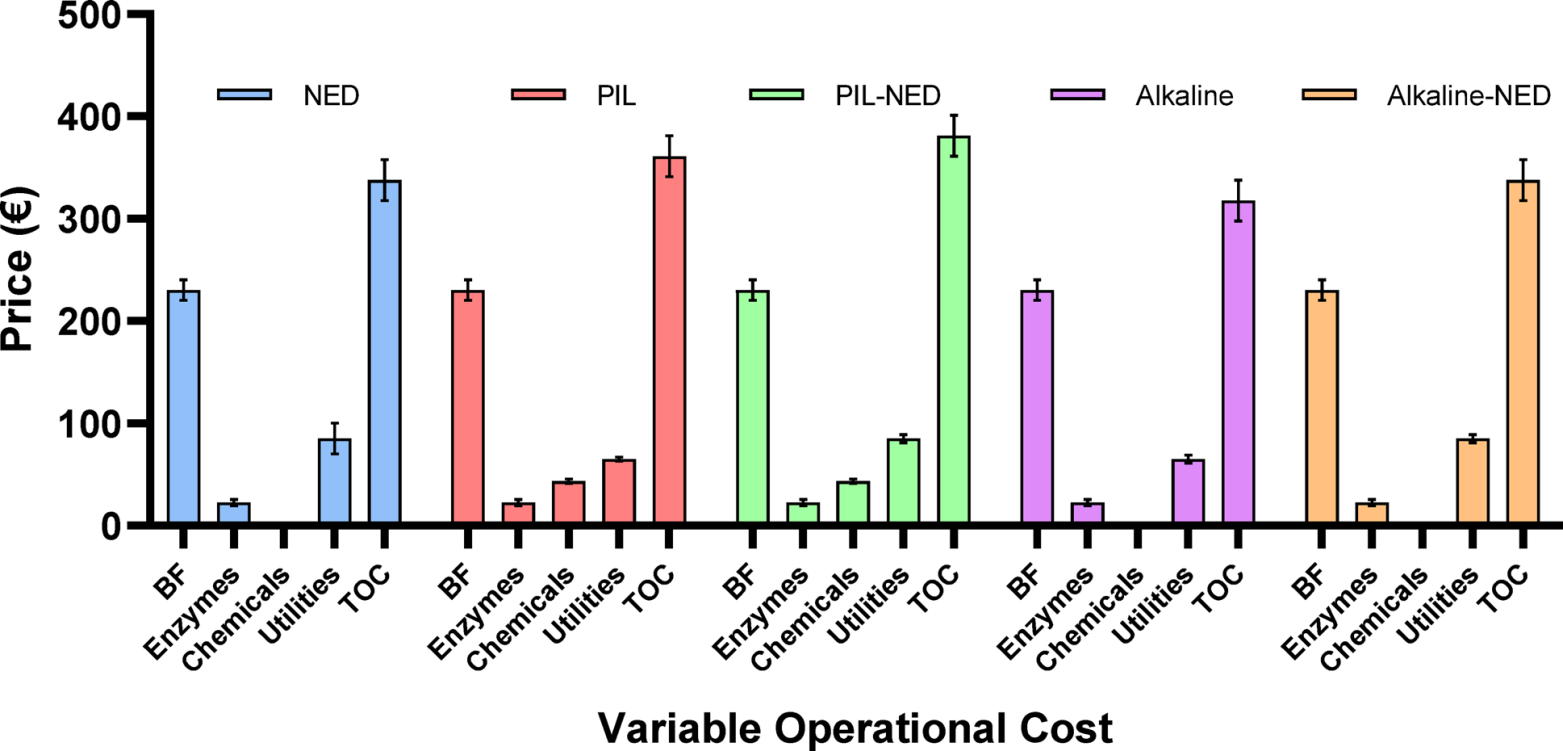
Total variable operating cost of different pretreatments processes. BF: biomass fractionation; TOC: total operating cost

A preliminary cost analysis of the raw material costs required to produce sugars, cellulose pulp, furfural, and lignin using different pretreatments is presented in Table 3. It is critical to recognize that this cost analysis solely encompasses raw material expenses and does not include the costs associated with equipment, purification, downstream processing, and packaging (Fig. 7). Therefore, a direct comparison with full commercial market prices is not appropriate for this laboratory-scale study. Sensitivity analysis regarding solvent recycling and impurity impacts was informed by recent literature, while an in-depth solvent analysis was not conducted herein. Nevertheless, a high-level cost comparison provides initial insights into the process’s economic potential. For context, the approximate commercial prices of key products were considered: furfural, sugars, cellulose pulp, and lignin (data gathered from the recent literature, as shown in Table 3). The production cost was calculated based on one tonne of aspen biomass pretreatment conditions, which yielded different amounts of products, as presented in the mass-energy balances. It is important to note that optimizing the reaction conditions and scaling up the process can significantly increase or decrease the yields while reducing or increasing the production costs (Klein-Marcuschamer et al. 2011a, b; Climent Barba et al. 2022). Given that the estimated costs are derived from lower-Technology Readiness Levels (TRL) laboratory production.

Recently, Cheng et al. (2019) highlighted the cost associated to the first-generation biomass with the lowest fixed capital costs, ranging from 0.01 to 0.13 $ kg^−1^ feedstock, and have production costs ranging from 0.22 to 0.55 $ kg^−1^ sugar while for the cellulosic crops the fixed capital and production costs are higher, ranging from 0.02 to 1.10 $ kg^−1^ feedstock and 0.10 to 3.37 $ kg^−1^ sugar, respectively. Similarly, Baral and Shah (2016) asses the techno-economic feasibility of a commercial-scale Ionic Liquids (IL) pretreatment for a 113 million liter/year (30 million gal/year) cellulosic biorefinery and identify operational targets for process improvement. They acknowledged that the IL pretreatment to be economically competitive, > 97% IL recovery, ≤ $1/kg IL cost, and > 90% waste heat recovery is necessary. Also, Klein-Marcuschamer et al. (2011a, b) proposed a comprehensive sensitivity analysis and presents the most significant areas in terms of cost savings/revenue generation that must be addressed before IL pre-treatment can compete with other, more established, pre-treatment technologies. Altogether, few studies perform an early-stage assessment of potential revenue against operational costs before estimating large capital expenditures (Rodrigues Gurgel da Silva et al. 2018; Tschulkow et al. 2020; Bhatia et al. 2021). This analysis further provides a risk assessment for lab-oriented technologies with a TRLs of 1–2.

**Fig. 8:**
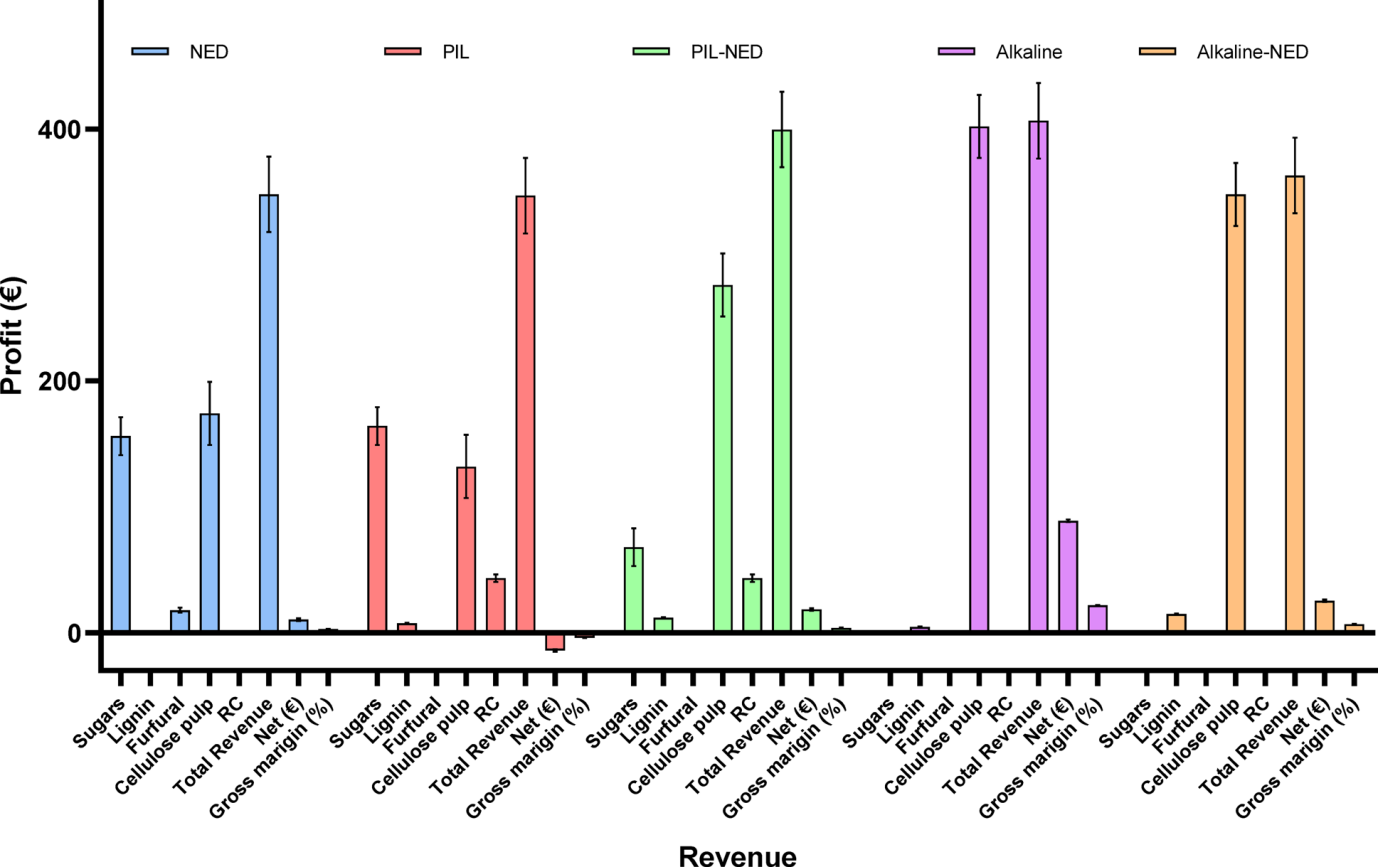
Cost analysis of different pretreatment methods and their revenue per tonne of aspen biomass

Results suggested that the pretreatment of aspen wood, sugars and recovered cellulosic pulp were identified as the primary revenue drivers, contributing approximately 90% of the total estimated income (Fig. 8). Furfural and lignin constitute the remaining revenues. The high gross margin, particularly for the recovered hemicellulose could significantly enhance the economic viability of PIL-NED pretreatment. Conversely, utility costs (water and electricity), feedstock, and enzymes were the major components of operating expenses, accounting for 25–70% of the total for specific pretreatment processes (Fig. 7). This was followed by the costs of the PIL, and solvents. The biomass cost can have approximately 30% lower harvesting costs (via direct chipping instead of baling and not using high quality log whilst replacing it with paper quality wood) (Hepner et al. 2021). Therefore, the utilization of aspen wood is vital for the economic and technical sustainability of the proposed value chain. Similarly, the conceptual design of biorefineries and economic assessments usually involve inaccurate data to estimate capital costs (Solarte-Toro et al. 2021).

**Table 3:** Initial costs of consumables and products for evaluating the preliminary technoeconomic analysis

	Cost	Units	Source
*Input*			
Feedstock (Aspen)^a^	150 ± 8	€/tonne	Hepner et al. (2021)
Milling and grinding^b^	28 ± 2	€/tonne	Yang et al. (2025)
Pretreatment	52 ± 7	€/tonne	Tschulkow et al. (2020), Rooni et al. (2021), Dezashibi et al. (2025)
PIL (optimistic case of 99.9% solvent recovery)^c^	13 ± 1	€/tonne	Bhatia et al. (2021), Dezashibi et al. (2025)
Process water^d^	0.04 ± 0.01	€/L	Rodrigues Gurgel da Silva et al. (2018)
MetGen cellulose blend (Suno)^e^	150 ± 18	€/tonne biomass	Hämäläinen et al. (2021)
EtOH (99.9% solvent recovery)^f^	0.8 ± 0.1	€/L	Patel et al. (2025), Bangalore Ashok et al. (2018)
Electricity	0.2	€/kWh	Krzywnicka and Barner (2025)
*Output*			
Sugars (glucose and xylose)^g^	400 ± 23	€/tonne	Dessbesell et al. (2019)
Lignin (heating value)	150 ± 9	€/tonne	Xu et al. (2024)
Furfural	1800 ± 35	€/tonne	Patel et al. (2025), Zang et al. (2020)
Cellulose pulp	600 ± 40	€/tonne	Yang et al. (2025)

^a^Price includes an estimated 10% discount on aspen wood. In Estonia 2024 price for high quality aspen log was 61.56 €/m^3^. Dried to 10% moisture 1 m^3^ weighs about 430 kg then 1 tonne would cost about 143 ± 10 €/tonne. Similarly, if we don’t use high quality log and replace it with paper quality wood then it is about 1/3 cheaper ending up at 104 €/tonne

^b^Price includes energy consumption for milling and grinding

^c^For the PIL base case, 100 g of biomass and 300 g IL loading were used

^d^A 60% (w/w) and ~ 90% anti-solvent loading and recovery were assumed

^e^Recommended enzymatic dosage (0.15% w/w biomass loading)

^f^A solvent recovery rate of 99% was assumed, it is critical to note that a rate of 90% along with the impact of solvent impurities significantly alters the feasibility of the cost analysis

^g^EU market price for white sugar was assumed however prices can be volatile and are suspected to inflation

A promising strategy for the efficient valorization of aspen biomass involves combining NED and PIL pretreatments. This hybrid approach leverages the complementary strengths of each pretreatment: NED effectively deconstructs the biomass, whereas PIL facilitates subsequent lignin extraction and fractionation with high purity, which is critical for high-value applications of lignin. Many authors Jiju et al. (2025), Babaeipour et al. (2025) and Österberg et al. (2020) has established solvent fractionation as a viable method for producing lignin-based nanoparticles, which are promising for bio-based coatings, resins, and adhesives. Given that the PIL-NED pretreatment extracts lignin with ethanol and yields a lower molecular weight product compared to the technical lignins, future work should explore its direct integration for producing colloidal lignin nanoparticles (CLPs), particularly for developing sustainable coatings in packaging applications.

Preliminary analysis suggests that alkaline extraction and alkaline-NED (e.g., a Kraft process simulation) could enhance profitability by approximately 22% and 7% (Fig. 8), respectively, compared to using NED, PIL, or PIL-NED pretreatment alone. This enhancement was primarily attributed to increased cellulose recovery. The Kraft process, utilized by the majority of pulp industries, employs NaOH to produce cellulose pulp and generates energy by burning lignin (Argyropoulos et al. 2023). However, for high-value lignin valorization rather than mere combustion, the extraction of a purer lignin stream earlier in the process via PIL-NED is advantageous over conventional methods (Jiju et al. 2025). Liao et al. (2020) and Sun et al. (2018) presented the potential of lignin-derived platform chemicals utilizing advanced biorefinery concepts. However, alkaline pretreatment extracts lignin in the form of black liquor, the presence of impurities often limits its application to low-value applications as we addressed in this study.

To maximize cost-efficient biomass utilization, future work must investigate two integrated aspects: optimizing furfural co-production from the hemicellulose stream in the PIL-NED process, and enhancing the enzymatic hydrolysis following PIL or alkaline-assisted pretreatment. While, this study did not demonstrate the feasibility of furfural isolation, fermentation and economic challenges for solvent recovery and product purification. Future research on efficient separation techniques is required to improve the economic viability of integrated PIL biorefining processes. Consequently, this study provides essential mass balance data that aligns with core biorefinery design criteria, where the integration of pretreatment, hydrolysis, solvent recycling, and fermentation must be optimized to maximize the biomass value. Similarly, this study does not provide a full techno-economic analysis, it offers crucial insights into how the choice of biomass and pretreatment technologies fundamentally shapes processing outcomes.

## Conclusions

The selection of a pretreatment method for LCB fractionation involves a critical trade-off between the operational cost and the potential for integrated biorefinery valorization of the lignin fraction. Conventional alkaline and alkaline-assisted thermochemical methods are effective in producing a high-yield cellulose streams, as presented in this study. Furthermore, these processes typically result in severe condensation and degradation of lignin, rendering it unsuitable for anything beyond low-value combustion for process heat production. Consequently, the economic viability of these pathways relies almost exclusively on the value of the cellulosic fraction, with the value proposition of lignin remaining minimal.

In contrast, advanced pretreatment systems, such as PIL-assisted technologies, are designed to enhance the functionality of lignin during fractionation. This “lignin-first” approach prioritizes the extraction of a high-quality depolymerized lignin stream that can be funneled into high-value applications, such as renewable polymers for packaging, carbon fibers, or fine chemicals in cosmetics, rather than simply being burned. Cost analyses indicate that the higher capital and operational expenses associated with these selective pretreatments create significant economic challenges. Therefore, the industrialization of PIL-based or similar fractionation technologies is intrinsically linked to the development of stable and profitable markets for valorized lignin production applications. Pretreatment selection is no longer just a question of sugar yield, but a strategic decision dictated by the potential for integrated product streams and the successful commercialization of lignin-derived products.

## Supplementary Information

Below is the link to the electronic supplementary material.

Supplementary Material 1

## Data Availability

The data supporting this article have been included as part of the Supplementary Information.
