# Supplementary material for: Maximizing Populus tremula biomass conversion: synergistic pretreatment effects on sugars release and lignin recovery
Source: Bioresour Bioprocess. 2026 Apr 15;13(1):54. doi: 10.1186/s40643-026-01040-5 (PMC13079260; doi:10.1186/s40643-026-01040-5)
Supplement: Supplementary file 1 — Supplementary Material 1 [file 40643_2026_1040_MOESM1_ESM.docx]

*Supplementary Information for*

**Maximizing *Populus tremula* Biomass Conversion: Synergistic Pretreatment Effects on Sugars Release and Lignin Recovery**

Sharib Khan ^a^, Vahur Rooni ^a^, Daniel Rauber ^b, c^, Nikki Sjulander ^a^, Markus Gallei ^b, c^, Christopher W. M. Kay ^d, e^, Sabarathinam Shanmugam ^a,^ *, Timo Kikas ^a,^ *

^a^ Chair of Biosystems Engineering, Institute of Forestry and Engineering, Estonian University of Life Sciences, Tartu, Estonia

^b^ Polymer Chemistry, Saarland University, Campus C4.2, 66123 Saarbrücken, Germany

^c^ Saarene, Saarland Center for Energy Materials and Sustainability, Saarland University, 66123 Saarbrücken, Germany

^d^ Physical Chemistry and Chemistry Education, Saarland University, Campus B2.2, 66123 Saarbrücken, Germany

^e^ London Centre for Nanotechnology, University College London, 17-19 Gordon Street, London WC1H 0AH, UK

* Correspondence: [sabarathinam.shanmugam@emu.ee](mailto:sabarathinam.shanmugam@emu.ee), [timo.kikas@emu.ee](mailto:timo.kikas@emu.ee)

**Table S1:** The chemical composition (Cellulose, hemicellulose and ash) of the Populus tremula before and after different pretreatments.

| **Conditions** | **Cellulose (w/w %)** | **Hemicellulose (w/w %)** | **Ash (w/w %)** | **Moisture (w/w %)** |
| --- | --- | --- | --- | --- |
| Untreated aspen (As-received basis) | 53.82 ± 0.10 | 16.90 ± 0.30 | 1.17 ± 0.10 | 3.55 ± 0.24 |
| NED | 69.70 ± 1.64 | 9.66 ± 2.02 | 1.34 ± 0.32 | - |
| PIL | 75.98 ± 0.70 | 13.78 ± 2.57 | 1.25 ± 0.43 | - |
| PIL-NED | 86.66 ± 2.67 | 6.02 ± 3.77 | 0.28 ± 0.25 | - |
| Alkaline | 85.35 ± 1.01 | 0.55 ± 1.51 | 0.66 ± 0.42 | - |
| Alkaline-NED | 96.42 ± 1.94 | 0.00 ± 0.00 | 0.16 ± 0.04 | - |

***Table S2:*** *Effect of pretreatments on the sugars yield.*

| **Conditions** | **Glucose (g)** | **Xylose (g)** | **Arabinose (g)** | **Mannose (g)** | **Cellobiose (g)** |
| --- | --- | --- | --- | --- | --- |
| Untreated Aspen (As-received basis) | 5.34 ± 3.23 | 0.97 ± 0.11 | - | 0.17 ± 0.29 | - |
| NED | 30.50 ± 1.42 | 8.51 ± 1.01 | 0.05 ± 0.09 | 0.65 ± 0.13 | - |
| PIL | 30.22 ± 3.80 | 10.13 ± 0.57 | - | 0.50 ± 0.03 | 0.53 ± 0.18 |
| PIL-NED | 15.33 ± 2.67 | 1.69 ± 0.15 | - | - | - |
| Alkaline | 0.15 ± 0.26 | - | - | 0.15 ± 0.14 | 0.55 ± 0.05 |
| Alkaline-NED | 0.22 ± 0.18 | - | - | - | - |

**Table S3**: Effect of different pretreatments on delignification percentage and lignin yield.

| **Conditions** | **Lignin (w/w)** | **Delignification (%)** | **Recovered lignin (g)** |
| --- | --- | --- | --- |
| Untreated Aspen (As-received basis) | 13.89 ± 0.28 | - | - |
| NED | 11.51± 0.60 | - | - |
| PIL | 6.44 ± 0.60 | 51.12 ± 0.91 | 5.4 ± 0.80 |
| PIL-NED | 4.14 ± 0.19 | 68.42 ± 1.19 | 7.8 ± 0.28 |
| Alkaline | 8.74 ± 0.66 | 34.49 ± 1.41 | 3.03 ± 0.3 |
| Alkaline-NED | 1.94 ± 0.58 | 85.68 ± 1.85 | 9.50 ± 0.37 |
